# Fabrication and Characterization of Hybrid Hole Transporting Layers of Organotin (IV) Semiconductors within Molybdenum Oxide/Poly(3,4-ethylenedyoxithiophene) Polystyrene Sulfonate Matrices

**DOI:** 10.3390/polym14194143

**Published:** 2022-10-03

**Authors:** María Elena Sánchez Vergara, César Raúl Monzón González, José Ramón Álvarez Bada, Leon Hamui, Cecilio Álvarez Toledano

**Affiliations:** 1 Facultad de Ingeniería, Universidad Anahuac Mexico, Avenida Universidad Anáhuac 46, Col. Lomas Anáhuac, Huixquilucan 52786, Mexico; 2 Instituto de Química, Universidad Nacional Autónoma de Mexico, Circuito Exterior s/n. C.U., Delegación Coyoacán, Ciudad de México 04510, Mexico

**Keywords:** PEDOT:PSS, molybdenum oxide, organotin(IV) semiconductor, optical properties, hole transport layer

## Abstract

The hybrid film of molybdenum oxide (MoO_3_) and poly(3,4-ethylenedyoxithiophene) polystyrene sulfonate (PEDOT:PSS) is a promising candidate for use as hole transport layer (HTL) in low-cost devices. A fast, controllable and economic process was used to fabricate high-performance HTLs by adding organotin (IV) semiconductors to the MoO_3_/PEDOT:PSS films. These hybrid films were fabricated by spin-coating and the MoO_3_/PEDOT:PSS-organotin (IV) complex films were characterized by infrared spectroscopy, scanning electron microscopy (SEM) and atomic force microscopy (AFM). Some mechanical and optical properties of the hybrid films were obtained and, to electrically characterize the hybrid films, hetero-junction glass/ITO/MoO_3_/PEDOT:PSS-organotin (IV) complex/Ag devices were prepared. Regarding the mechanical properties, the films have high plastic deformation, with a maximum stress of around 40 MPa and a Knoop hardness of 0.14. With respect to optical behavior, the films showed high transparency, with optical gap values between 2.8 and 3.5 eV and an onset gap of around 2.4 eV, typical of semiconductors. Additionally, the films in their respective devices show ambipolar and ohmic behavior with small differences depending on the substituent in organotin (IV) semiconductors. The MoO_3_/PEDOT:PSS matrix defines the mechanical behavior of the films and the tin complexes contribute their optoelectronic properties.

## 1. Introduction

**Figure 1:**
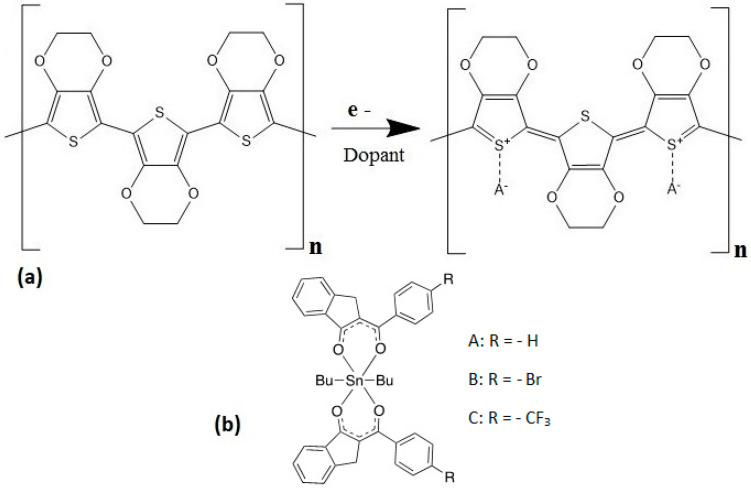
Structures of (**a**) PEDOT and (**b**) organotin (IV) complexes.

Semiconducting polymers have found important applications as materials for the production of optoelectronic devices, given their simple processability, low manufacturing cost, miniaturization capacity, high sensitivity and the possibility of molecular design through chemical synthesis [1,2]. In 1989, a polythiophene derivative, poly(3,4-ethylenedyoxithiophene) (PEDOT) was reported for the first time. Very soon, it acquired a prominent position among these polymers, due to its conductor properties. PEDOT is an intrinsic semiconductor, since its molecules are capable of conducting electricity. Its monomers are characterized by electron relocation and the polymer consists of long chains with conjugated bonds, which favors the formation of electronic orbitals extended throughout the chain, promotes free electron movement and keeps relocation within its monomers [3]. PEDOT also has a low band gap and shows excellent stability against atmospheric oxidation [4,5,6,7]. These properties have allowed PEDOT to be widely used as an antistatic layer or as a material for hole transport in optoelectronic devices [8]. On the other hand, PEDOT cannot act as a conductor on its own, but needs the presence of ions in its structure that favor the movement of electrical charges. That is, it needs to be subjected to a doping process and, due to the fact that PEDOT can transit rapidly and reversibly from doped to undoped state and vice versa by means of repetitive redox processes (see Figure 1a), it is a conductor polymer that can be both *p*- and *n*-doped [9]. All these properties make PEDOT very attractive for multiple applications that involve a proper selection of its counter-ion [10]. For example, PEDOT is not soluble in water but, when polymerized with poly(styrene sulfonate) (PSS), a form of PEDOT:PSS is obtained that can be dispersed in water. PSS acts as a *p*-type doping equilibrator during the polymerization, as it acts as an acceptor; PSS chains produce *p*-type doping of the PEDOT chains by oxidation. The PEDOT segments will remain in the oxidation state due to the reducing effect of the PSS segments [11]. The PEDOT rusty state (PEDOT^+^) is highly conductive, while its neutral form (PEDOT^0^) is less conductive. PSS acts as the dopant anion that PEDOT needs to be a conductor and permits the use of molding and printing techniques to obtain flexible films that are useful in electronics [12]. Currently, conductive PEDOT:PSS films are found as electronic components, because of their ease of manufacture and treatment in different geometries down to nano- and micrometric scale, as well as their flexibility. Examples of the many uses of PEDOT:PSS films include the one developed by Yemata et al. [13] with PEDOT:PSS films treated with hydrazine to enhance the Seebeck coefficient, the performance improvement of organic light-emitting diodes (OLEDs) and polymer solar cells (PSCs) achieved by Song et al. [14] by means of a treatment with solvent in buried PEDOT:PSS layers and the approach developed by Yeon et al. [15], which involved treating PEDOT:PSS conductor films with sodium dodecyl sulfate to manufacture stretchable fabric heaters.

One of the main advantages of using PEDOT:PSS involves the formation of ohmic contacts with metals. This polymer has a high work function of approximately 5 eV [16], which makes it a good choice for use as a hole-injector layer (HTLs). However, due to the hydrophilic nature of PEDOT:PSS, it is quite complicated to deposit on the hydrophobic photoactive layer surface [17]. The hygroscopic nature of PEDOT:PSS also has an adverse impact on device lifetime [18,19,20]. Devices based on PEDOT:PSS HTL degrade very fast when exposed to ambient air [18] and, because of its acidic nature, PEDOT:PSS can erode the ITO electrode [18,21,22,23,24] and affect device performance and reliability [18,23,25]. To circumvent these problems, some transition metal oxides, such as MoO_3_ [17,18,23,26,27], WO_3_ [17,18,23,28,29,30] and V_2_O_5_ [17,18,23,31,32], have been used to substitute PEDOT:PSS as HTLs [17,18,23]. Of these transition metal oxides, molybdenum oxide (MoO_3_), is rather promising and frequently employed, due to its non-toxicity and deep-lying electronic states with a large work function of 5.5–6.7 eV [17,18,33]. In terms of low-cost and large-area production, solution-processed MoO_3_ is preferred for use as HTL [18]. However, the low conductivity of MoO_3_ makes it necessary for a film to be used in devices, such as photovoltaic solar cells, which is a big challenge for most low-cost and large-area processing technologies [18]. Therefore, it is desirable to develop HTLs where the properties of MoO_3_ are combined with those of the PEDOT:PSS, in order to obtain stable films under service conditions that perform efficiently in charge-transport terms within optoelectronic devices. MoO_3_ has been less employed to modify PEDOT:PSS, such as HTL, to obtain the improved performance of OLEDs or PSCs. We report here the preparation and characterization of MoO_3_/PEDOT:PSS ink that can be deposited by spin-coating to form HTLs. The contribution of this work lies specifically in reporting and investigating the effect of organotin (IV) semiconductors derived from 2-hydroxybenzylidene-1-indanones in MoO_3_/PEDOT:PSS as a way to enhance its properties and photovoltaic and optoelectronic applications. The main interest areas of organotin complexes are polymer stabilizers [34,35], catalytic agents [36,37] and biological agents with cytotoxic, antioxidant and anti-inflammatory properties [38,39,40,41,42]. However, there are few reports on the photovoltaic and optoelectronic applications of thin films of organotin (IV) complexes [43,44], which is why we decided to expand these studies. Preliminary results of the study of organotin (IV) complexes of 2-hydroxybenzylidene-1-indanones derivatives as thin films showed electronic properties potentially useful in the production of optoelectronic devices [45]. Based on the above, we report in this work the structural, morphological, mechanical, optical and electrical properties of organotin (IV) complexes in MoO_3_/PEDOT:PSS matrices of HTLs. Different organotin (IV) complexes were used as dopants. In Figure 1b, it is shown that their structural difference is due to the substituents present in their periphery. In organic semiconductors, it is possible to modify their charge transport capacity with the presence of electro-attracting or electro-donating substituents. For this work, it was decided to study the effect of electro-attracting groups on the flow of electrons in tin (IV) complexes. The A-complex, with hydrogen substituents, is considered as the reference. The bromide radicals of B-complex are less electro-attractive than the CF_3_ radicals of the C-complex.

The MoO_3_/PEDOT:PSS-organotin (IV) complex films were deposited by using the spin-coating technique and their stability and topography were studied by infrared (IR) and atomic force microscopy (AFM), respectively. Additionally, morphological characteristics were investigated with a scanning electron microscope (SEM). Subsequently, the optical behavior and optical band gap of MoO_3_/PEDOT:PSS-organotin (IV) semiconductor films were investigated by UV-vis spectroscopy. Finally, heterojunction devices with the structure glass/ITO/MoO_3_/PEDOT:PSS-organotin (IV) complex/Ag were fabricated with each of the hybrid HTLs and their electrical behavior was later examined.

## 2. Materials and Methods

All reagents, solvents and the molybdenum trioxide/poly(3,4-ethylenedioxythiophene)-poly(styrenesulfonate) (MoO_3_/PEDOT:PSS) ink were obtained from commercial suppliers (Sigma-Aldrich, Saint Louis, MO, USA) and used without further purification. The organotin (IV) complexes were obtained according to procedures previously reported by some of the authors of this work [45]. The structure of these complexes can be seen in Figure 1b and the notation can be described as follows: A-complex: C_40_H_40_O_4_Sn, B-complex: C_40_H_38_Br_2_O_4_Sn and C-complex: C_42_H_38_F_6_O_4_Sn. The MoO_3_/PEDOT:PSS-organotin (IV) complex films were deposited by spin-coating; a Smart Coater 200 equipment (Laurell Technologies Corporation North Wales, PA, USA) was used. The dispersion used for the manufacture of the films consisted of 5 mL of MoO_3_/PEDOT:PSS dispersion in ethanol and 2-propanol and 5 mg of A, B and C organotin (IV) semiconductors. A saturated dispersion was then generated with every organotin (IV) complex. The mixture MoO_3_/PEDOT:PSS-organotin (IV) complex was dispersed using a G560 shaker of Scientific Industries Vortex-Genie (Bohemia, New York, NY, USA). The dispersion was later deposited on the substrate and the equipment was operated for one time at an angular speed of 300 rpm for a spin time of 30 s and an acceleration of 272 rpm/s. Hybrid films were deposited on *n*-type silicon wafers (polished on a single side), Corning glass and indium tin oxide (In_2_O_3_·(SnO_2_)_x_; ITO)-coated glass slides. The Corning glass and the glass–ITO substrates were at first sequentially washed in an ultrasonic bath with dichloromethane, methanol and acetone. The silicon substrates were washed with a “p” solution (10 mL HF, 15 mL HNO_3_ and 300 mL H_2_O) in order to remove surface oxide. After deposition, the films were dried at 85 °C for 10 s on a hot plate. AFM measurements of the films on silicon substrates were performed in contact mode with a static tip and with a Nanosurf Naio microscope (Nanosurf, Liestal, Switzerland). Morphological characteristics were investigated with a ZEISS EVO LS 10 scanning electron microscope (Carl Zeiss AG, Jena, Germany). The FTIR spectroscopic analysis was performed for the compounds as KBr pellets and for the films on a silicon substrate using a Nicolet iS5-FT spectrometer (Thermo Fisher Scientific Inc., Waltham, MA, USA), at a wavelength range of 4000 to 500 cm^−1^. The UV-vis spectroscopy of the films on glass was performed in the 200–1100 nm wavelength range, on a UV-Vis 300 Unicam spectrophotometer (Thermo Fisher Scientific Inc., Waltham, MA, USA). For the electrical characterization of the films, hybrid devices were fabricated using an ITO anode and a silver cathode: glass/ITO/MoO_3_/PEDOT:PSS-organotin (IV) complex/Ag. For this evaluation, a programmable voltage source, a sensing station with a lighting controller circuit from Next Robotix (Comercializadora KMox, S.A. de C.V., Mexico City, Mexico) and an auto-ranging Keithley 4200-SCS-PK1 pico-ammeter (Tektronix Inc., Beaverton, OR USA) were employed.

## 3. Results and Discussion

### 3.1. Morphological and Mechanical Characterization of Hybrid Films

**Figure 2:**
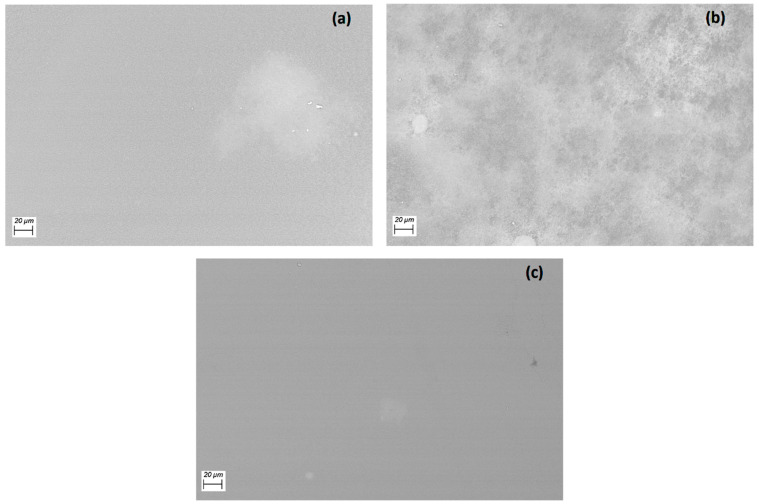
SEM images of (**a**) MoO_3_/PEDOT:PSS-A organotin (IV), (**b**) MoO_3_/PEDOT:PSS-B organotin (IV) and (**c**) MoO_3_/PEDOT:PSS-C organotin (IV) films.

**Figure 3:**
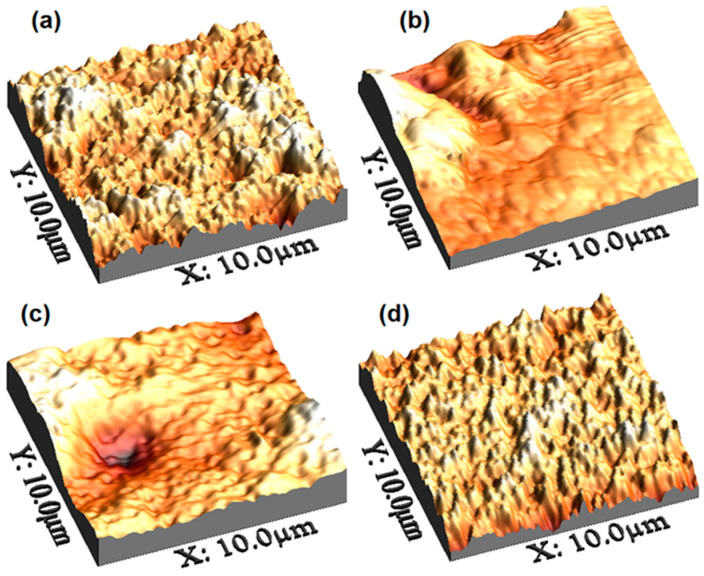
AFM images of (**a**) MoO_3_/PEDOT:PSS, (**b**) MoO_3_/PEDOT:PSS-A organotin (IV), (**c**) MoO_3_/PEDOT:PSS-B organotin (IV) and (**d**) MoO_3_/PEDOT:PSS-C organotin (IV) films.

Efficient charge transport through the HTL requires that the film be continuous and ordered. In the films deposited by spin-coating, amorphous dominos are formed that may lead to low-charge mobility. For this reason, SEM was carried out; Figure 2 shows the resulting micrographs at 750×. In all the films, a surface free of holes or imperfections that act as traps or scattering centers for electric charge transport is observed. Regarding homogeneity, the film with A-complex segregation of particles that form large clusters can be seen in Figure 3a. In the film with the B-complex, the phases corresponding to the dispersed heterojunction between the polymer, the MoO_3_ and the complex are evenly distributed. The film with the C-complex is uniform and this film is the one that could present the best optoelectronic and photovoltaic behavior.

To enhance the information corresponding to film morphology, it is important to analyze their topography, since the increase in the size of the grains and roughness, along with a good continuity in the films, is a good approach to improve the transport and the mobility of charges in devices, such as organic solar cells [46]. The topography of the films was studied through AFM. Figure 3 shows the image for a 10 μm × 10 μm area and the RMS (Root Mean Square) and Ra (Roughness Average) are provided in Table 1 for the pristine film formed by MoO_3_/PEDOT:PSS (Figure 3a) and for the hybrid films. The lowest roughness was found in the pristine film and the highest roughness was obtained in the film with A-organotin (IV) complex. In this case, a film with an irregular topography formed by particle segregation was observed, with individual particles of sizes smaller than 1 μm and by areas with valleys in which there are apparently no particles of the tin complex (Figure 3b). Similar results were obtained for the film with B-organotin (IV) complex (Figure 3c), which, although presenting a considerably lower roughness than the film with A-complex, also shows an irregular topography due to a heterogeneous distribution of the particles in the MoO_3_/PEDOT:PSS matrix. Finally, in the film made from the MoO_3_/PEDOT:PSS-C organotin (IV) complex, roughness is not much larger than in the pristine film. In addition, a uniform topography is found with grains of sizes less than 1 μm and distributed throughout the MoO_3_/PEDOT:PSS matrix (Figure 3d) [46]. It is important to consider that the spin-coating operating parameters for all film deposition, as well as the stoichiometric relationship between its precursors MoO_3_/PEDOT:PSS and the organotin (IV) complex, are similar in all cases. From the above, it is considered that the effect of the organotin (IV) complex is significant in the topography and roughness of the films. The inclusion of organotin (IV) complex in MoO_3_/PEDOT:PSS reduces the segregation between PEDOT conductor and PSS insulator, with a reduction in the electrostatic attractive force between PEDOT and PSS [47]. By including the organotin (IV) complex in MoO_3_/PEDOT:PSS, the RMS is increased in MoO_3_/PEDOT:PSS–organotin (IV) complex films. The RMS increase in these films may be due to the thinning effect after the interaction of organotin complexes in PEDOT:PSS. As organotin in the dispersion forms, it readily interacts with the PEDOT and PSS chains and a denser morphology is formed in MoO_3_/PEDOT:PSS–organotin (IV) complex films [47], with the C-organotin (IV) complex favoring a more uniform topography. Furthermore, according to the SEM results, this film presents a homogeneous morphology, which can favor (i) an adequate interface with the electrodes and (ii) the efficient transport of charges in photovoltaic and optoelectronic devices.

Some mechanical properties of the films were evaluated, considering a maximum applied force of 990 N. Under this condition, the maximum stress (σ_max_), the unitary deformation (ε) and the Knoop microhardness (HK) of the MoO_3_/PEDOT:PSS-organotin (IV) complex films were determined and the results are presented in Table 1. While the stress and hardness values are consistent with this type of film and its deposition process, it is important to remember that the high deformation obtained is related to a high-plastic region affecting the mechanical behavior in the films. Therefore, under service conditions, it is advisable to keep them free from mechanical loads or stress. As expected, the film with the C-complex was the one that endured the highest stress, as it had a higher homogeneity and a better dispersed heterojunction between the PEDOT:PSS, the MoO_3_ and the tin complex. This was the film with the smallest deformation and the greatest hardness. It is important to mention that, although the films with complexes A and B are heterogeneous, their mechanical parameters do not show significantly different values with respect to the film with C-complex. These results highlight the importance of PEDOT:PSS and MoO_3_ as matrix materials. Just as the mechanical behavior of the films relies on the matrix, it may be expected that the tin complexes contribute their optical and electrical properties to the behavior of these hybrid films.

**Figure 4:**
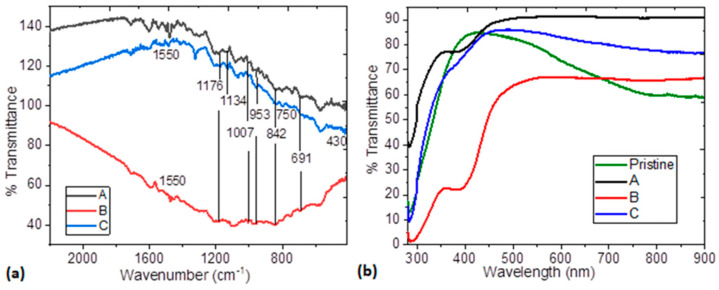
(**a**) IR spectra of MoO_3_/PEDOT:PSS-organotin (IV), (**b**) Transmittance spectra of pristine MoO_3_/PEDOT:PSS and MoO_3_/PEDOT:PSS-organotin (IV) complex films.

Low chemical stability is one of the main problems in organic semiconductor films. In order to evaluate the chemical stability of the hybrid-film MoO_3_/PEDOT:PSS–organotin (IV) complex, IR spectroscopy was carried out after deposition and after evaluating its optical and electrical properties. It is important to verify that, during deposition, no degradation of the organotin (IV) complexes and the polymer matrix has taken place. In the spectra shown in Figure 4a, the typical bands in PEDOT:PSS can be identified. The C-O-C bending vibrations in the ethylenedioxy group occur between 1172 and 1134 cm^−1^, while C-S-C stretching vibrations in the thiophene ring occur between 955 ± 2 and 691 cm^−1^ [48,49]. The 1007 cm^−1^ band is assigned to the O–S–O symmetric stretching mode in PSS and C–H angular deformation of the aromatic ring in PSS gives a band at 842 cm^−1^ [15,48]. Regarding the organotin (IV) complexes, the IR spectrum shows an intense absorption band in the 1550–1590 cm^−1^ region that was assigned to C=O and C=C stretching vibrations with considerable mixing. The presence of bands in a range of 730–750 cm^−1^ was attributed to Sn–C vibrations [29,45]. Finally, the stretching bands that occur around 430 cm^−1^ were attributed to Sn–O bonds [29,45]. From the IR spectroscopy results, it is concluded that the components in the hybrid films do not suffer degradation during film formation. Additionally, Table 2 shows the IR spectroscopy signals obtained from the films after the evaluation of their properties. When comparing these results with those obtained for the original deposited films, it is observed that there are no significant changes in them, which suggests an adequate chemical stability in the MoO_3_/PEDOT:PSS–organotin (IV) complex films.

### 3.2. Evaluation of Optical and Electrical Properties

**Figure 5:**
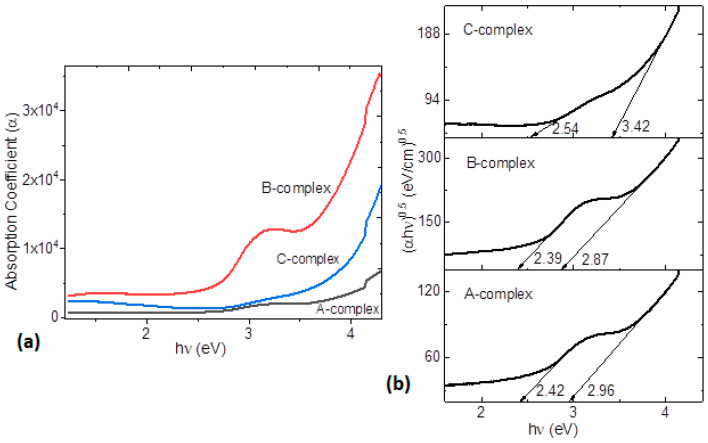
(**a**) Spectral behavior of absorption coefficient and (**b**) variation of (αhν)^1/2^ with hν of MoO_3_/PEDOT:PSS-organotin (IV) complex films.

Figure 4b shows the transmittance as a function of wavelength of pristine MoO_3_/PEDOT:PSS and the MoO_3_/PEDOT:PSS-organotin (IV) complex. According to Zhou et al. [23], MoO_3_ and the PEDOT:PSS matrix have little effect on the transmittance of films and the most important effect is exerted by the organotin (IV) complex. While the pristine film has high transmittance in a small region of the spectrum, the films with the tin complexes are transparent from 450 nm onwards. The highest transmittance, corresponding to 90%, is found in the film with the A-complex, followed by 86% transmittance in the film with the C-complex, although the transmittance in this film drops to 76% at longer wavelengths. Finally, the film with the B-complex presents transmittance of 66% which, despite lower than that obtained in the film with the C-complex, remains uniform at longer wavelengths. It seems that the higher transmittance in the film with A-complex may be due to the lack of electro-attracting substituents in its structure. Nevertheless, the three films with organotin (IV) complex seem to have applicability as transparent electrodes in optoelectronic devices [50,51]. Optically transparent HTLs are important in organic electronic applications, such as OLEDs and PSCs, because they allow the photoactive layer to produce the photocurrent intensity [52]. According to Hilal et al. [52], the MoO_3_/PEDOT:PSS-organotin (IV) films would be useful as HTLs in photonics and organic electronics applications. Figure 5a exhibits the absorption coefficient α as a function of incident energy hν for the MoO_3_/PEDOT:PSS-organotin (IV) complex films. It can be observed that the α-values are of the order of 10^4^ for the films with the A and C-complexes and 10^5^ for the film with the B-complex. The absorption spectra of the MoO_3_/PEDOT:PSS-B organotin (IV) complex film shows a red shift in the absorption edge and a higher intensity of the absorption peaks related to the complex type.

In many organic semiconductors, it is customary to study the type of optical transitions, as well as the value of the optical band gap, because most charge transitions in organic semiconductors happen between the HOMO and the LUMO, with the band gap representing the region between them. From UV-vis spectroscopy, the value of the HOMO-LUMO optical interval, E_opt_, is attributed to the lower-energy transition that takes place by absorption of a photon. Bardeen et al. [53] related the band gap of semiconductor films to its absorption coefficient (α) and incident photon energy (hν) through (αhν) = A(hν−E_opt_)^r^, where E_opt_ is the optical band gap and A depends on the type of transition; r is a power that takes the value 2 for indirect electronic transitions related to amorphous semiconductor films [53]. Figure 5b shows (αhν)^1/2^ as a function of hν. The E_opt_ value can be calculated by extrapolating a tangent line to the hυ axis in (αhν)^1/2^ = 0. The plots for the films illustrate the onset gap, E_g_^onset^, which corresponds to the onset of optical absorption and formation of a bound electron-hole pair, or “Frenkel exciton” [54,55]. The second transition obtained for all the films is E_opt_ [54,55,56]. Both values of E_g_^onset^ and E_opt_ are shown in Figure 5b. The film with the B-complex is the one with the lowest values and, therefore, could be the best semiconductor followed by the film with the A-complex. The difference between the band gaps for these films is due to the presence of the substituent in the structure of the complexes. These differences are more significant in the film with the C-complex, which only provides a reliable value for E_opt_. This value turns out to be greater than those obtained for the other hybrid films. The CF_3_ substituent definitely exerts a marked influence on the C-organotin (IV) complex and apparently decreases the semiconductor behavior of the material. CF_3_ is a strong electro-attracting group, pulling electronic density in its direction which can polarize and hinder charge transport between the molecules of the complex and at the complex-matrix interfaces. Nevertheless; in the film with the neutral A-complex, charge transport is no better than in the film with B-complex. The presence of the bromide in this complex, with an electro-attracting capability greater than A and less than C, promotes intermolecular and intramolecular electronic transitions, without generating charge polarization. On the other hand, it is important to mention that the value of 3.42 eV obtained for the film with the C-complex is similar to the E_opt_ obtained for thin films in PEDOT:PSS, with heptacoordinated organotin (IV) complexes having substituents that can be both electro-attractors and electro-donors [57,58], as previously studied by some authors of this work. With respect to hybrid films with a PEDOT:PSS matrix and graphene and organotin (IV)-based semiconductor complexes with electro-attracting substituents, such as chloride [59], and for the organotin Schiff bases in spun films applied in organic solar cells [60], the E_opt_ obtained for the films with the A and B-complexes presents similar values and, in all cases, less than that of the film with the C-complex. Apparently, this result is related to the small size of the bromide radical, in addition to its electro-attracting character. This result is important because, on one hand, there are few recent studies related to organotin complexes as semiconductor films and, on the other hand, if these films are to be used in optoelectronic or photovoltaic devices, it is important that the organotin complex has a radical or electro-attracting substituent of small size in its structure.

**Figure 6:**
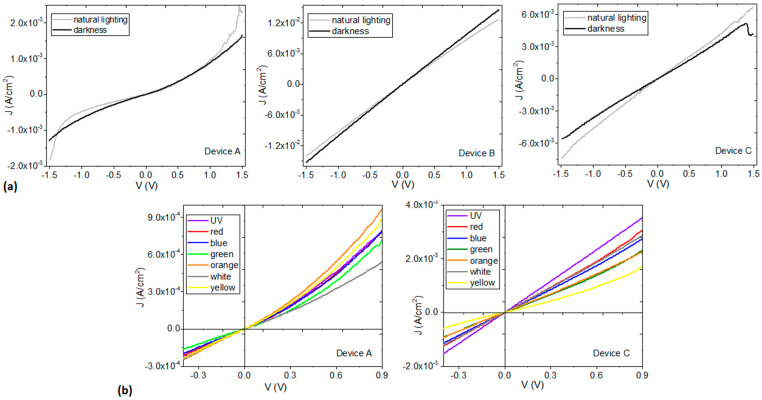
J-V curves from ITO/MoO_3_/PEDOT:PSS-organotin (IV) complex/Ag devices under (**a**) natural lighting/darkness conditions and (**b**) different lighting conditions.

In order to investigate the electrical properties of the MoO_3_/PEDOT:PSS–organotin (IV) complex films, simple devices were fabricated and their current-voltage (I-V) characteristics were measured under natural lighting conditions and in darkness. In this work, devices of only one layer were manufactured in which the hybrid film MoO_3_/PEDOT:PSS-organotin (IV) complex was deposited over the anode, which, in this case, corresponded to the ITO conductive film. The circuit closes with the silver cathode, which is deposited above the hybrid film. The electrical behavior is shown in Figure 6a. It can be seen, from the current density–voltage curves (J-V), that the three films in their respective devices show ambipolar behavior. It seems that these films can transport both electrons and holes, depending on the applied electric field. On the other hand, the devices with the films of the B and C complexes show an ohmic behavior. Moreover, for the B-complex device, the graphs obtained from measurement under conditions of natural illumination are essentially the same as those obtained under darkness conditions, which may limit the operation of this film in OLED or PSC applications, where optical semiconductors capable of promoting charge generation in the films are required. Nevertheless, it is worth noting that the B-complex film is the one with the largest charge transport, in agreement with the smaller E_opt_ value that it has when compared to the other films (see Figure 5b). It is also worth mentioning that the values of J, for the three devices, are within the same order of magnitude as the ones obtained by Wang et al. [17], by Shao et al. [18] and by Zhou et al. [23] for optimized MoO_3_/PEDOT:PSS hybrid HTL. Regarding the A-complex device, the film showed a different electrical behavior from those of the other devices. Although there was also an increase in the value of the transported J with increasing V, there was less charge transport as a result of the high-polarity bromine substituent in the structure of the Sn complex (see Figure 1b) and the high film roughness (see Table 1) [17]. The electrical parameters for the devices are shown in Table 1, where the conductivity and short circuit current density (J_sc_) values were obtained for darkness and natural illumination conditions. It can be observed, for the darkness condition, that the highest conductivity was obtained by the B complex, supporting the previous results, while the C and A complexes present a lower conductivity. The latter may affect the series resistance of a PSC and device efficiency. The observed values are between 10^−7^ and 10^−6^ S cm^−1^ and are comparable to the literature [17,61,62]. For natural illumination conditions, these conductivity values are increased in all the complexes, but the largest variation is observed for the B complex (1.2 × 10^−7^ S cm^−1^), followed by the C complex. These results prove the effect of the organotin (IV) complex on the absorption and charge transport when considering their use in PSC. On the other hand, the current density values for both illumination conditions (Table 1) are quite similar for all the devices but higher for the B complex, indicating that the matrix effect is predominant. These values are increased under natural lighting conditions, as much as 1.8 × 10^−7^ A/cm^2^ in variation (A complex). The smallest variation is observed in the B complex (~0.9 × 10^−7^ A/cm^2^), while C complex has an intermediate value (~1.3 × 10^−7^ A/cm^2^). Due to the nature of the hybrid films and supported by the literature, it is expected that these values present a very small degradation with time, ensuring device stability for optoelectronic applications [18].

As the devices with films A and C are the ones showing a different behavior under conditions of natural illumination and darkness, they were also analyzed under different irradiation conditions in the portion of the electromagnetic spectrum between infrared and ultraviolet (see Figure 6b). This was done in order to determine their electrical transport capacity and their possible use in OLEDs or PSCs, where photon absorption becomes a necessary attribute. In the corresponding J–V curve, it is found that, at voltages larger than 0.3 V, the type of radiation incident on the devices modifies charge transport. The largest J for the A-complex device is obtained with red-light exposure and the smallest J with white light, whereas the largest J for the C-complex device is obtained with UV radiation and the smallest J with yellow light. The presence of the CF_3_ substituent in the Sn complex (see Figure 1b) is a determining factor in the electrical behavior change of the device.

**Figure 7:**
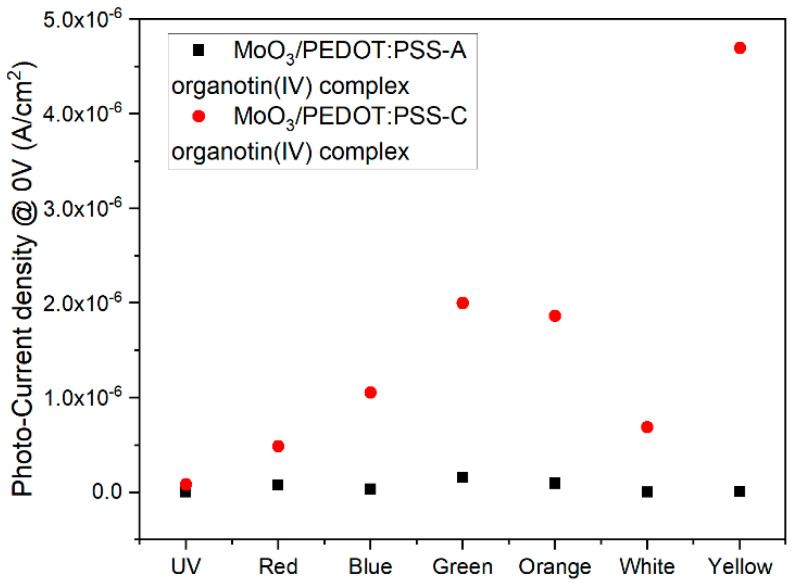
Photocurrent density at 0 V for ITO/MoO_3_/PEDOT:PSS-organotin (IV) complex/Ag devices under different lighting conditions.

To evaluate the device characteristic output due to the lighting effect, the photo-current density at 0 V was obtained and is presented in Figure 7. The photocurrent variation is indicative of the device charge carrier generation and collection efficiency. The last is also related to the charge carrier mobility within the film and contact extraction capability. In this case, Figure 7 compares A and C complexes, where a variation in the photo-current density values and the plot behavior is observed. First, a light-color-induced change in the photocurrent density between 1.8 × 10^−9^ and 1.6 × 10^−7^ A/cm^2^ for A complex is observed, while between 8.4 × 10^−8^ and 4.7 × 10^−6^ A/cm^2^ for C complex is observed. The plot behavior is different by comparing the devices, indicative of the complex effect on the optoelectronic parameters. The larger photocurrent density values may be related to C-complex film homogeneity and lower roughness. The highest photocurrent is observed for the green and yellow-light colors, for A and C complexes, respectively, which can be considered a consequence of the aforementioned optical band gap variation. By comparing the photo-current density for the green color, due to the organotin complex, a variation of approximately an order in magnitude is observed. Moreover, according to these results, the presence of substituents in the organotin structure leads to an ohmic behavior, although the MoO_3_/PEDOT:PSS matrix turns out to be responsible for the mechanical strength of the film.

## 4. Conclusions

Hybrid films based on PEDOT:PSS with MoO_3_ were fabricated and reinforced in their optoelectronic behavior with the introduction of organotin (IV) complexes, Complex A: C_40_H_40_O_4_Sn, Complex B: C_40_H_38_Br_2_O_4_Sn and Complex C: C_42_H_38_F_6_O_4_Sn. The films have high plastic deformation and, in general, mechanical properties related to the MoO_3_/PEDOT:PSS matrix. According to UV-vis spectroscopy, there is a transparent behavior and the highest absorption coefficient is found in the film with the bromide substituent (B-organotin (IV) complex). This film and the film with the A-organotin (IV) complex (which has no substituent in its peripheral structure), show clearly defined onset and optical band gaps, but the film with the C complex (CF_3_ substituent) only allows for a reliable determination of the optical gap. Regarding the electrical behavior, the three films show an ambipolar and mainly ohmic behavior. The MoO_3_/PEDOT:PSS–organotin (IV) complex films are promising candidates for use as hole transport layers. 

## Figures and Tables

**Table 1:** Roughness, mechanical and electrical parameters of the hybrid films.

Film	RMS (nm)	Ra (nm)	Thickness (μm)	Area (m^2^)	σ_max_ (MPa)	ε	HK	Conductivity Darkness (S cm^−1^)	Conductivity Natural Lighting (S cm^−1^)	J_sc_ Darkness (A/cm^2^)	J_sc_ Natural Lighting (A/cm^2^)
Pristine MoO_3_/PEDOT:PSS	33.83	26.99	0.75	2.226 × 10^−5^	40	0.987	0.112	-	-	-	-
MoO_3_/PEDOT:PSS-A organotin (IV) complex	396.2	300.9	1.30	2.477 × 10^−5^	40	0.980	0.144	1.22 × 10^−7^	1.40 × 10^−7^	−5.62 × 10^−7^	−3.82 × 10^−7^
MoO_3_/PEDOT:PSS-B organotin (IV) complex	40.05	30.19	1.18	2.234 × 10^−5^	44.3	0.979	0.159	1.03 × 10^−6^	1.15 × 10^−6^	−5.17 × 10^−7^	−4.26 × 10^−7^
MoO_3_/PEDOT:PSS-C organotin (IV) complex	33.91	27.86	1.11	2.181 × 10^−5^	45.4	0.946	0.163	4.22 × 10^−7^	4.91 × 10^−7^	−5.63 × 10^−7^	−4.30 × 10^−7^

**Table 2:** Band positions and assignments of MoO_3_/PEDOT:PSS-organotin (IV) complex films.

Assignment	MoO_3_/PEDOT:PSS-A Organotin (IV) Complex	MoO_3_/PEDOT:PSS-B Organotin (IV) Complex	MoO_3_/PEDOT:PSS-C Organotin (IV) Complex
C–O–C bending	1173, 1130	1172, 1130	1167, 1136
C–S–C stretching	955, 690	945, 701	945, 673
O–S–O symmetric stretching	1011	1022	1019
C–H angular deformation	838	843	841
C=O stretching C=C stretching	1550–1598	1550–1592	1547–1592
Sn–C	727–740	730–750	738–750
Sn–O	434	437	437

## Data Availability

Not applicable.
